# Enhanced Object Detection Algorithms in Complex Environments via Improved CycleGAN Data Augmentation and AS-YOLO Framework

**DOI:** 10.3390/jimaging11120447

**Published:** 2025-12-12

**Authors:** Zhen Li, Yuxuan Wang, Lingzhong Meng, Wenjuan Chu, Guang Yang

**Affiliations:** 1 Ocean College, Jiangsu University of Science and Technology, Zhenjiang 212003, China; justwyx@stu.just.edu.cn (Y.W.); cindy66920@163.com (W.C.); 2 Institute of Software Chinese Academy of Sciences, Beijing 100190, China; yangguang@iscas.ac.cn

**Keywords:** image enhancement, object detection, CycleGAN, feature fusion, deep learning

## Abstract

Object detection in complex environments, such as challenging lighting conditions, adverse weather, and target occlusions, poses significant difficulties for existing algorithms. To address these challenges, this study introduces a collaborative solution integrating improved CycleGAN-based data augmentation and an enhanced object detection framework, AS-YOLO. The improved CycleGAN incorporates a dual self-attention mechanism and spectral normalization to enhance feature capture and training stability. The AS-YOLO framework integrates a channel–spatial parallel attention mechanism, an AFPN structure for improved feature fusion, and the Inner_IoU loss function for better generalization. The experimental results show that compared with YOLOv8n, mAP@0.5 and mAP@0.95 of the AS-YOLO algorithm have increased by 1.5% and 0.6%, respectively. After data augmentation and style transfer, mAP@0.5 and mAP@0.95 have increased by 14.6% and 17.8%, respectively, demonstrating the effectiveness of the proposed method in improving the performance of the model in complex scenarios.

## 1. Introduction

In application scenarios such as intelligent security, autonomous driving, and unmanned monitoring, object detection technology is playing an increasingly critical role [1]. However, real-world environments are often affected by complex lighting conditions, adverse weather, and target occlusions, which severely compromise detection accuracy and may lead to the failure of object recognition algorithms. Therefore, enhancing the performance of object detection under complex and failure-prone conditions is essential to ensure the stability and practicality of recognition systems and algorithms.

In recent years, object detection techniques have made significant progress and are generally categorized into two-stage and single-stage detectors. Two stage detectors such as Faster R-CNN [2]. achieve high-precision detection through two stages of candidate region generation and classification regression, which are suitable for scenarios that require high accuracy. Single stage detectors such as YOLO series [3] and SSD [4] achieve fast detection through end-to-end architecture and are suitable for tasks with strong real-time requirements.

Despite this progress, object detection in complex environments still faces numerous challenges [5]. Lighting issues such as backlighting, glare, or shadows can reduce image contrast or cause overexposure, impairing the model’s ability to accurately delineate object boundaries. Harsh weather conditions—including rain, snow, fog, and dust—introduce image blur, occlusion, and color distortion, which hinder the extraction of effective texture and edge features by the model [6]. Conventional object detection models often rely on clean and structured data distributions, while real-world data are highly variable and complex. This discrepancy limits the models’ ability to learn representative and discriminative features, thereby weakening their generalization across different scenarios. Addressing these issues solely by training on large-scale annotated real-world datasets is costly and still insufficient to cover all possible failure conditions. Therefore, enhancing the robustness and adaptability of detection systems through data augmentation and architectural optimization is urgently needed.

To tackle the challenges of object detection in failure-prone environments, researchers have explored various data augmentation strategies. Sakaridis et al. [7] constructed the Foggy Cityscapes dataset by synthetically adding fog to the original Cityscapes images, enabling the training and evaluation of object detection models under foggy conditions. Dai et al. [8] introduced the Nighttime Driving dataset, which contains real-world nighttime traffic scenes and provides high-quality samples for nighttime detection tasks. Yan et al. [9] proposed the RainGAN model, which utilizes generative adversarial networks (GANs) to synthesize rainy scenes, thereby improving the robustness of object detection models under rainy weather conditions. Pan et al. [10] proposed DICNet, a Retinex-based network integrating image decomposition, illumination enhancement, and color restoration, achieving superior low-light enhancement with noise suppression and natural color recovery. Wang et al. [11] proposed a two-stage low-light image enhancement network called SBC-Net, which integrates semantic information and discrete brightness curves to significantly improve enhancement performance. Xue et al. [12] proposed an underwater image enhancement algorithm combining color correction and contrast enhancement, which effectively addresses color distortion and low contrast issues through gray world assumption and guided filtering techniques. Jiang et al. [13] proposed a single-stage GAN with multi-scale feature aggregation and boosting-based denoising to enhance low-light images while preserving details and reducing noise. Lee et al. [14] proposed a model called ScoreGAN, which is trained using the Inception score, an evaluation metric for GANs, as a rough guide for generator training. Sabry et al. [15] proposed a sketch-based image retrieval approach using Information Maximizing Generative Adversarial Networks (InfoGAN), which enables unsupervised learning of salient visual features and improves retrieval performance for large-scale sketch datasets. Lin et al. [16] proposed URDRN, an unrolled rain-guided detail recovery network that decomposes single-image deraining into rain extraction and detail restoration sub-tasks, using a rain attention map to guide fine-grained background reconstruction while preserving texture fidelity. Xu et al. [17] adopted RegCGAN to achieve corresponding imbalanced data learning. Yang et al. [18] proposed MultiScaleConv-YOLO (MSConv-YOLO), which enhanced YOLOv8s for UAV small-target detection by integrating a multi-scale convolution module, WIoU v3 loss, and a high-resolution detection head, significantly improving accuracy and recall. Tan et al. [19] presented MidState-YOLO-ED, an enhanced YOLOv10-based model that integrated YOLOv8’s detection head, efficient multi-scale attention (EMA), and a lightweight C2f-Dual module to improve accuracy and efficiency in retail self-checkout product recognition.

Despite the extensive research efforts outlined above, existing approaches face fundamental limitations that restrict their effectiveness in complex environmental scenarios. First, limited global context modeling: standard CycleGAN generators rely predominantly on local convolutional operations with restricted receptive fields, failing to capture long-range spatial dependencies essential for generating coherent atmospheric effects across the entire image. Second, training instability under small-batch scenarios: the widely adopted batch normalization (BN) is sensitive to batch statistics, causing style shift and mode collapse when training with limited GPU memory. To address this issue without relying on batch statistics, spectral normalization stabilizes the training of generative adversarial networks by constraining the spectral norm of each weight matrix in the discriminator, thereby controlling the Lipschitz constant of the discriminator function [20]. Third, structural distortion due to inadequate semantic constraints: the original cycle consistency loss operates at the pixel level and cannot effectively preserve high-level semantic structures, resulting in geometric distortions during style transformation. To enforce higher-level semantic consistency, perceptual constraints based on deep features are required. Johnson et al. [21] demonstrated that pixel-wise losses fail to capture perceptual differences, motivating the use of feature-level constraints for improved structural fidelity.

Similarly, current feature fusion strategies in the YOLO series exhibit notable deficiencies when applied to complex environments. First, semantic degradation during feature propagation: although PANet introduced a bottom-up path to enhance FPN, it still does not fully address the detail loss or degradation when features are passed between levels, nor the semantic gap between non-adjacent hierarchical features. The sequential fusion approach causes semantic information to be diluted during cross-layer transmission. Second, insufficient attention to spatial dependencies: mainstream channel attention mechanisms such as SE-Net focus exclusively on channel-wise recalibration while neglecting spatial attention, limiting the model’s ability to distinguish targets from cluttered backgrounds. CBAM addresses this by combining channel and spatial attention, yet its integration into detection frameworks remains suboptimal. Third, scale-insensitive bounding box regression: conventional IoU-based loss functions treat all target scales uniformly, failing to provide adaptive gradient signals for objects of varying sizes, which compromises localization accuracy for multi-scale targets commonly encountered in complex environments.

These limitations collectively hinder the performance of object detection systems in complex environments. A collaborative optimization framework that simultaneously addresses data-level augmentation quality and model-level feature extraction capability is therefore urgently needed.

To address these challenges, we propose a collaborative optimization approach from both data and model perspectives. For data augmentation, an enhanced image generation method based on an improved CycleGAN [22] is designed to simulate image samples in complex scenarios. The key improvements include: (1) the introduction of dual self-attention mechanisms before and after the ResNet blocks in the generator to enhance long-range dependency modeling; (2) the replacement of batch normalization with spectral normalization to improve training stability; and (3) the addition of a feature consistency loss to reduce artifacts and preserve structural integrity.

On the model side, a novel object detection framework, AS-YOLO, is developed based on YOLOv8. The main innovations include: (1) the integration of a channel–spatial parallel attention mechanism [23] to enhance feature focus capability; (2) the adoption of the AFPN structure [24] to improve multi-scale feature fusion; and (3) the use of the Inner_IoU loss function [25] to strengthen the model’s generalization ability. Experimental results demonstrate that the proposed method improves the detection accuracy and stability, achieving gains of 1.5% and 0.6% in mAP@0.5 and mAP@0.95, respectively.

The proposed CycleGAN and YOLO-based models were independently trained and tested on a custom multi-object dataset tailored for the application environment. Comparative experiments were conducted against several state-of-the-art methods to verify the effectiveness and superiority of the proposed approach under challenging conditions. Furthermore, ablation studies were performed to assess the individual contributions of each improvement strategy.

Building on these components, a collaborative optimization framework that combines the improved CycleGAN and AS_YOLO models was constructed. Comprehensive performance evaluations were carried out on the augmented dataset. The experimental results show that mAP@0.5 and mAP@0.95 have increased by 14.6% and 17.8%, respectively, proving that this method has strong object detection performance and robustness in complex environments. It also highlights its application value and deployment potential in complex environments.

## 2. Improved Style Transfer Algorithm Based on CycleGAN

Although traditional image enhancement methods can improve visual quality in certain scenarios, they often rely heavily on manually designed rules and fail to adapt to diverse weather conditions, resulting in insufficient realism of the generated images. The root cause lies in their use of fixed mathematical transformation strategies, which cannot effectively represent the complexity and variability of real-world weather. For instance, RGB-based synthetic fog methods typically apply Gaussian blur to smooth the image, but fail to simulate key physical properties such as light attenuation and scattering in foggy scenes, nor can they accurately capture the spatial variability between dense and light fog. Moreover, these methods require manual parameter tuning, significantly increasing implementation complexity. More critically, traditional approaches struggle to capture real-world lighting, color, and texture characteristics, particularly dynamic variations, due to their limited understanding of global environmental context.

To address these limitations, this paper proposes an improved style transfer method based on Cycle-Consistent Generative Adversarial Networks (CycleGAN). Leveraging an end-to-end unsupervised learning framework, the proposed method can autonomously learn complex lighting features, color distributions, and texture patterns from images, as well as diverse style transformation rules. This not only reduces the complexity of parameter tuning but also enhances the realism of the generated images in complex environmental conditions.

### 2.1. Dual Self-Attention Mechanism Module

In deep learning, the self-attention mechanism establishes dynamic dependencies among elements within a sequence, enabling adaptive modeling of sequential data. The core of self-attention lies in computing the relevance between each element and all others to capture long-range dependencies [26]. For style transfer tasks, incorporating self-attention into CycleGAN significantly enhances the model’s ability to model global contextual information, thereby improving semantic consistency during style transformation. Unlike recurrent neural networks (RNNs), which process data sequentially, self-attention employs a fully parallel computation strategy, thereby increasing both model expressiveness and training efficiency.

CycleGAN typically adopts U-Net or ResNet as the generator, but the limited receptive field of convolutional layers hinders the modeling of global features and may lead to visual artifacts. To overcome this, we introduce a dual self-attention mechanism into the ResNet-based generator:

A self-attention module is embedded before the ResNet blocks to utilize global context for optimizing residual learning and improving transformation quality;

Another self-attention module is added after the ResNet blocks to strengthen correlations among local features, making weather style transfer more coherent and visually natural.

The self-attention module follows the formulation proposed by Vaswani et al. Given an input feature map $F\in R^{C\times H\times W}$, it is first reshaped to $F^{\prime }\in R^{C\times N}$ where N=H×W. The query, key, and value projections are computed using 1 × 1 convolutions:

(1) $$Q=W_{Q}F^{\prime },\;K=W_{K}F^{\prime },\;V=W_{V}F^{\prime },$$

where $W_{Q},\;W_{K},\;W_{V}\in R^{C^{\prime }\times C}$ are learnable projection matrices with $C^{\prime }=\frac{C}{8}=32$. The attention output is computed as

(2) $$Attention(Q,K,V)=softmax(\frac{QK^{T}}{\sqrt{d_{k}}})V,$$

where *d_k_* = 32 is the scaling factor. We employ 8 parallel attention heads to capture diverse spatial dependencies. The computational overhead remains moderate since self-attention operates on bottleneck feature maps rather than high-resolution inputs.

This design allows for multi-scale long-range dependency modeling, thereby improving semantic consistency in style transfer without significantly increasing computational overhead.

Additionally, to address style shift problems caused by batch normalization (BN) during small-batch training, we replace BN with Spectral Normalization (SN). SN constrains the spectral norm of weight matrices to satisfy the Lipschitz constraint:

(3) $${\bar{W}}_{SN}(W)=W/\sigma (W),$$

where σ(W) denotes the largest singular value of *W*. This normalization ensures ${\left\|W\right\|}_{sp}\leq 1$, providing theoretical guarantees for stable GAN training by preventing gradient explosion and vanishing.

We selected SN over alternative normalization strategies based on the following considerations: (1) BN statistics depend on batch composition and cause style shift with small batches; (2) Instance Normalization (IN) normalizes per-instance but may wash out fine-grained style information; (3) Layer Normalization (LN) is designed for sequential data and less effective for convolutional features. In contrast, SN operates on weights rather than activations, preserving style details while ensuring training stability.

**Figure 1:**
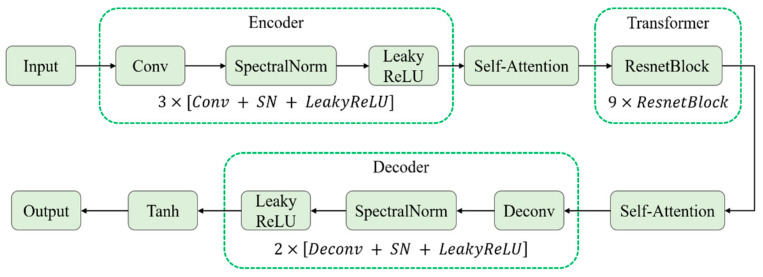
Generator Network with Improved Self-Attention Mechanism Integration.

As shown in Figure 1, the improved ResNet generator follows an “Encoder–Transformer–Decoder” architecture: The encoder consists of three convolutional blocks (3×[Conv+SN+LeakyReLU]) to extract local weather features; The transformer comprises nine ResNet blocks (9×ResnetBlock) for enhanced global feature representation; The decoder includes two deconvolutional blocks (2×[Deconv+SN+LeakyReLU]) to reconstruct the target style image. Experimental results show that the collaborative optimization of dual self-attention mechanisms and spectral normalization significantly improves the realism of generated images in terms of lighting, color, and texture, better aligning with the characteristics of real complex environments.

### 2.2. Feature Consistency Loss Function

The effectiveness of feature-space constraints over pixel-wise approaches has been consistently demonstrated across multiple domains. Pixel-wise losses process each pixel independently and fail to capture spatial dependencies and inter-pixel relationships, which limits their ability to preserve semantic structures and global coherence [27]. In contrast, feature consistency losses leverage high-level representations from pretrained convolutional networks, where deeper layers encode rich semantic information and global structural understanding [28]. In single-image super-resolution, Rad et al. [29] showed that replacing the uniform perceptual loss with semantically targeted feature-space losses (boundary loss for edges + texture loss for background) yields sharper edges and more realistic textures, and was preferred by users over pixel-wise MSE or the vanilla perceptual loss.

To enhance the stability and generation quality of CycleGAN in weather style transfer tasks, this study introduces a Feature Consistency Loss during training to improve structural coherence and suppress artifacts in the generated images [30].

While the original CycleGAN leverages a cycle consistency loss to constrain style transformations and ensure visual plausibility, its pixel-level constraints fail to capture high-level semantic features. As a result, the style conversion process may suffer from structural distortions, texture degradation, or even mode collapse. To address these issues, a feature consistency loss is incorporated to measure the similarity between input and generated images in high-level feature space, extracted from intermediate layers of deep neural networks. This enhances the model’s capacity to preserve both global context and local details.

Feature consistency loss is typically computed using intermediate features from a pretrained VGG network or the discriminator. The basic formulation is as follows:

(4) $$L_{\mathrm{feat}}=\frac{1}{N}\sum_{i=1}^{N}{∥\phi (x_{i})-\phi (G(x_{i}))∥}_{2}^{2},$$

Here, *x_i_* denotes the input image, $G(x_{i})$ is the corresponding generated image from the generator *G*, ϕ(·) represents features extracted from a pretrained network, *N* is the batch size, ${∥\cdot ∥}_{2}$ is the Euclidean norm for feature distance.

Feature Extraction Network: We employ VGG-19 pretrained on ImageNet as the feature extractor, following the practice established by Johnson et al. [31] for perceptual loss computation. As shown in Table 1, features are extracted from three layers at different semantic levels.

To ensure that structural information is preserved across different feature levels, the feature consistency loss can also be extended to a multi-scale weighted form:

(5) $$L_{\mathrm{feat}}=\sum_{m}\lambda_{m}\cdot \frac{1}{N}\sum_{i=1}^{N}{∥\phi_{m}(x_{i})-\phi_{m}(G(x_{i}))∥}_{2}^{2},$$

where *m* denotes the index of different feature layers, $\phi_{m}(\cdot )$ is the feature extractor at layer *m*, and $\lambda_{m}$ is the weight assigned to the corresponding layer.

To balance structural preservation in early training with stylistic refinement in later stages, we employ a cosine annealing schedule for the layer weights:

(6) $$\lambda_{m}\left(t\right)=\lambda_{m}^{base}\times \frac{1+cos\left(\frac{\pi t}{T}\right)}{2}+\lambda_{m}^{min},$$

where *t* is the current epoch, *T* is the total number of training epochs, $\lambda_{m}^{base}$ is the initial weight, and $\lambda_{m}^{min}$ is the minimum weight.

The final objective loss function of the generator is defined as

(7) $$L_{total}=L_{GAN}+\lambda_{\mathrm{cycle}}L_{\mathrm{cycle}}+\lambda_{\mathrm{feat}}\times \sum_{m}\lambda_{m}\times \frac{1}{N}\sum_{i=1}^{N}{∥\phi_{m}\left(x_{i}\right)-\phi_{m}\left(G\left(x_{i}\right)\right)∥}_{2}^{2},$$

Specifically, the feature consistency loss utilizes deep features extracted from either a pretrained VGG network or the discriminator’s internal layers and applies mean squared error (MSE) to enforce similarity in feature space between input and generated images. Compared to pixel-wise L1 or L2 losses, this approach more effectively captures global structural information during weather style transfer, reducing the risk of blurriness and color distortion caused by over-reliance on low-level pixel information.

To further stabilize training, a multi-scale feature extraction strategy is adopted, computing consistency loss at shallow, intermediate, and deep levels to ensure structural coherence across all semantic layers. Additionally, to prevent over-constraining the style generation process, a dynamically weighted loss scheme is employed: during early training stages, higher weights are assigned to maintain global structure, while in later stages, the focus shifts toward enhancing stylistic realism and fine-grained details.

**Figure 2:**
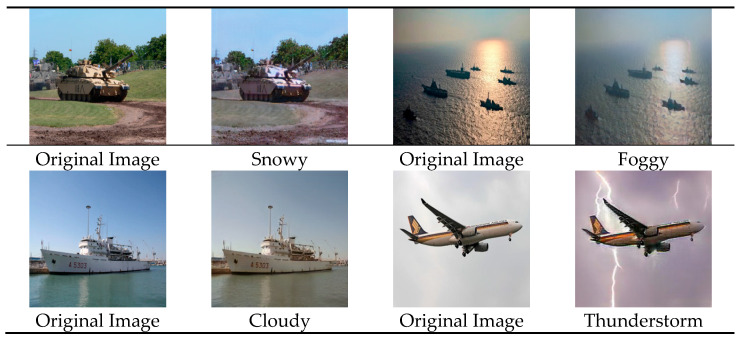
Improved CycleGAN Simulation Results.

In summary, the introduction of feature consistency loss significantly improves the structural fidelity of images generated by the improved CycleGAN, effectively suppresses artifacts, and enhances the quality and stability of weather style transfer outputs. The partial implementation example results are shown in Figure 2.

## 3. Improved YOLOv8 for Object Detection in Complex Environments

**Figure 3:**
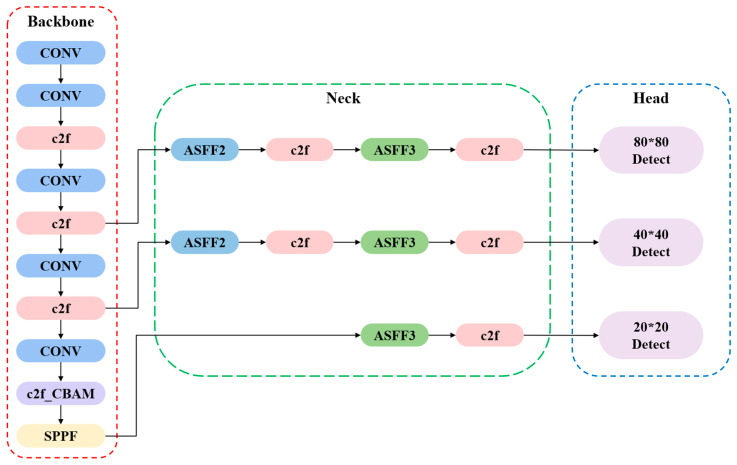
AS_YOLO model structure.

To enhance the performance of YOLOv8 in complex environments, this paper proposes a progressive object detection framework named AS-YOLO (Asymptotic Structure of YOLO), as illustrated in Figure 3.

The model achieves performance gains through three key innovations:

(1) Integration of the CBAM attention module into the backbone, leveraging a parallel channel–spatial attention mechanism to improve the model’s ability to distinguish targets from complex backgrounds;

(2) Replacement of the conventional PANet [31] with an Asymptotic Feature Pyramid Network (AFPN), which employs a hierarchical progressive fusion strategy to alleviate semantic degradation in multi-scale feature transmission;

(3) Introduction of an adaptive Inner-IoU loss function, whose dynamic adjustment mechanism significantly enhances the model’s adaptability to target scale variations and environmental complexity.

Experimental results demonstrate that AS-YOLO substantially outperforms the baseline model in terms of detection accuracy and robustness under challenging scenarios, making it particularly well-suited for challenging application scenarios such as object detection in complex environments.

### 3.1. The c2f_CBAM Module

In complex environments, targets often exhibit similar colors, textures, or shapes to the background, or are obscured by occlusions such as smoke or other objects, which makes detection difficult. Such conditions increase the complexity of features that detection algorithms must process, thereby imposing greater computational demands on the model. CBAM (Convolutional Block Attention Module) is a lightweight and widely applicable module that effectively enhances feature representation by combining channel [32] and spatial attention [33] mechanisms. It adaptively reweights feature maps based on channel importance, guiding the network to focus more precisely on target regions while suppressing background noise in cluttered scenes.

**Figure 4:**
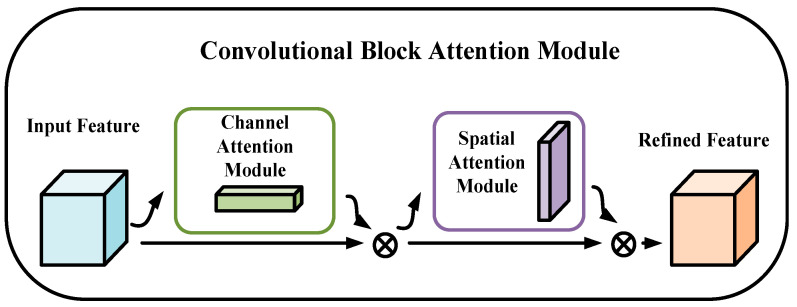
CBAM structure diagram.

By learning inter-channel relationships and spatial dependencies, CBAM enhances the network’s ability to adapt to complex environmental conditions and improves overall robustness. Its structure is shown in Figure 4.

The approximate calculation process of CBAM can be divided into two stages: channel attention and spatial attention. The specific process is as follows:

(1) Channel Attention Calculation

Given an input feature map $F\in R^{C\times H\times W}$, the channel attention map Mc(F) is computed as follows:

(8) $$\begin{array}{c} Mc(F)=\sigma (MLP(AvgPool(F))+MLP(MaxPool(F)))\\ \;=\sigma (W_{1}(W_{0}(F_{\mathrm{avg}\;}^{c}))+W_{1}(W_{0}(F_{max}^{c}))) \end{array},$$

where $W_{0}\in R^{C/r\times C}$ and $W_{1}\in R^{C+C/r}$ are the weights of the fully connected layers, *Avgpool* and *Maxpool* denote global average pooling and global max pooling operations, respectively, and MLP represents a shared two−layer fully connected network. σ is the sigmoid activation function.

(2) Spatial Attention Calculation:

The refined feature map *F*′ is obtained by performing pixel−wise multiplication between the input feature map *F* and the channel attention map Mc(F). Then, the spatial attention map $Ms(F^{\prime })$ is computed as

(9) $$\begin{array}{c} M_{s}\left(F^{\prime }\right)=\sigma (f^{7\times 7}([AvgPool(F^{\prime });MaxPool(F^{\prime })]))\\ \;=\sigma (f^{7\times 7}([F_{avg}^{s};F_{max}^{s}])) \end{array},$$

where $f^{7\times 7}$ denotes a convolution operation with a 7 × 7 kernel. Finally, the output feature map $F^{\prime \prime }$ is obtained by pixel-wise multiplication of $Ms(F^{\prime })$ and $F^{\prime }$.

**Figure 5:**
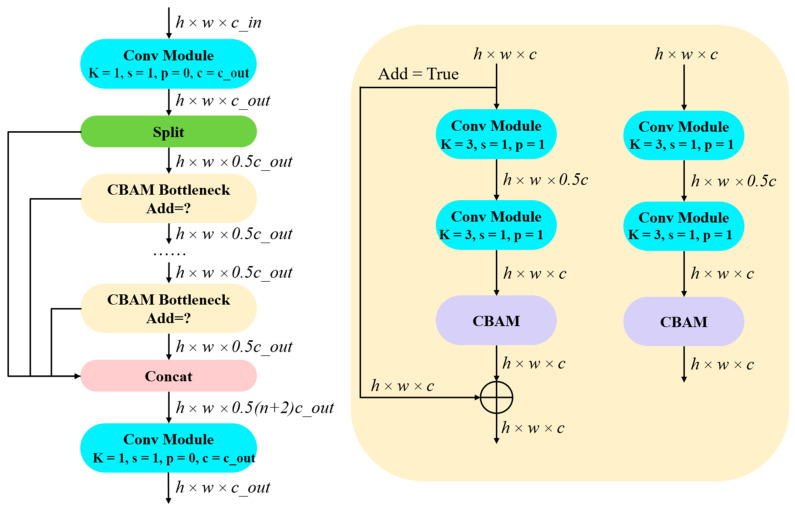
c2f_CBAM structure diagram.

To reduce computational overhead, the CBAM module is integrated into the deeper layers of the backbone network. Since shallow feature maps have high spatial resolution, introducing CBAM at early stages would significantly increase computational cost. In contrast, deep features possess stronger semantic representation capabilities; thus, applying CBAM at these stages not only reduces the computational burden but also enhances discriminative feature learning. Figure 5 illustrates the structural differences in the c2f module before and after the integration of CBAM.

### 3.2. Asymptotic Feature Pyramid Network

**Figure 6:**
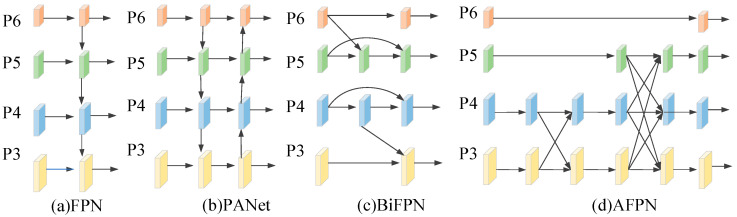
(**a**–**d**) reprents schematic diagram of using different feature fusion networks.

In complex environments, object detection is challenged by variations in imaging angles, detection distances, lighting conditions, and camouflage strategies, resulting in significant multi-scale target variability and increased detection difficulty. To address scale variation, most mainstream methods employ feature pyramid structures, such as the classical FPN [34]. YOLOv8 enhances FPN with PAFPN, introducing a bottom-up path to supplement high-level features with detailed low-level information. However, this may lead to degradation of shallow features. BiFPN [35] further improves deep feature fusion via repeated bidirectional connections, but at the cost of increased computational complexity and memory consumption due to additional links. In contrast, the Asymptotic Feature Pyramid Network (AFPN) used in this study optimizes cross-level fusion with the following advantages:

(1) Enhanced Information Interaction: AFPN strengthens fusion between non-adjacent layers, preserving high-level semantic features while maintaining low-level details, thereby reducing information loss during feature transmission.

(2) Computational Efficiency: Despite the seemingly more complex connectivity topology shown in Figure 6, AFPN achieves superior computational efficiency compared to BiFPN through several key architectural innovations. First, asymptotic fusion strategy: Rather than processing all feature levels simultaneously like BiFPN, AFPN employs a progressive fusion approach that starts with two adjacent low-level features and asymptotically incorporates higher-level features. This sequential processing significantly reduces the semantic gap between non-adjacent levels, enabling more efficient feature integration with fewer intermediate computations. Second, channel-dimension reduction: AFPN uses 1 × 1 convolutions to reduce the feature channel dimensions before fusion, significantly lowering computational overhead while preserving essential information. Third, lightweight structural design: The fusion nodes are designed with lightweight residual units and adaptive spatial fusion, which maintain representational power without excessive computational cost.

(3) Improved Robustness: The improved propagation and interaction mechanisms minimize feature degradation, leading to enhanced detection stability in complex scenarios.

As illustrated in Figure 6d, AFPN adopts a hierarchical progressive fusion strategy. It first integrates multi-resolution low-level features extracted by the backbone, then gradually incorporates semantically rich high-level features. Finally, a top-level fusion step enhances the overall feature representation capability.

To address feature conflicts during cross-scale fusion, AFPN incorporates the Adaptive Spatial Feature Fusion (ASFF) mechanism. ASFF dynamically assigns weights to features from different levels, enabling the model to learn the relative contributions of each layer automatically. This allows it to filter out redundant information and preserve only the most discriminative features for the detection task, thereby significantly improving both fusion efficiency and detection accuracy.

**Figure 7:**
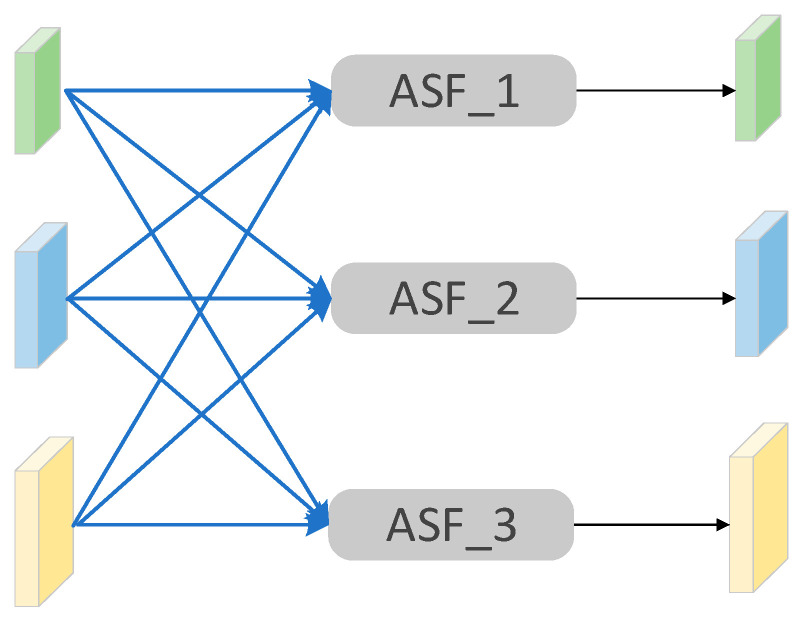
Adaptive spatial network architecture diagram.

As shown in Figure 7, ASFF integrates features from three levels. Each feature map is fed into its corresponding ASFF module (ASF_1, ASF_2, ASF_3), which selectively aggregates spatial information from all layers to produce an optimized output feature map.

The effectiveness of ASFF in improving fusion quality and detection accuracy stems from three fundamental mechanisms supported by recent research. First, spatial conflict resolution: ASFF addresses the inherent inconsistency problem in feature pyramids by learning to spatially filter conflictive information at each location. Unlike simple concatenation or summation approaches, ASFF enables the network to suppress contradictory features while preserving complementary information, thereby reducing the semantic ambiguity that typically degrades detection performance in multi-scale scenarios. Second, adaptive importance weighting: The mechanism dynamically computes spatial importance weights using softmax normalization, allowing features from different pyramid levels to contribute adaptively based on their relevance to the specific spatial context [36]. This data-driven approach ensures that at each spatial location, the most discriminative features dominate the fusion process while irrelevant or noisy features are automatically suppressed, leading to more robust and accurate object localization. Third, enhanced multi-scale feature fusion: By incorporating the ASFF module into the neck network, the module adaptively learns spatial weights for feature maps at different pyramid levels, enabling effective integration of semantic and detailed information across multiple scales [37].

### 3.3. Inner_IoU Loss Function

To address the challenges of multi-scale and multi-source data in object detection under complex environments, this study introduces the Inner-IoU loss function. This method employs a dynamic scaling factor to adjust auxiliary bounding boxes adaptively, allowing it to effectively handle scale variations caused by near–far alternation (i.e., large, medium, and small targets) as well as discrepancies across heterogeneous sensor data.

In contrast to traditional IoU-based metrics, Inner-IoU innovatively focuses on the alignment quality within the internal region of the bounding boxes, significantly enhancing detection sensitivity for small objects and non-standard aspect ratio targets.

The key insight behind Inner-IoU is that standard IoU loss exhibits diminishing gradients as predictions approach ground truth, which slows convergence in later training stages. By computing IoU on scaled auxiliary boxes, Inner-IoU modulates the gradient magnitude to accelerate convergence.

Consider a predicted box $B=(x_{c},y_{c},w,h)$ and ground truth box $B^{gt}=(x_{c}^{gt},y_{c}^{gt},w^{gt},h^{gt})$. The standard IoU gradient with respect to box width w is

(10) $$\frac{\partial L_{IoU}}{\partial w}=\frac{\partial (1-IoU)}{\partial w}=-\frac{\partial IoU}{\partial w},$$

For high-IoU samples (*IoU* → 1), this gradient approaches zero, causing slow convergence. Inner-IoU addresses this by introducing scaled auxiliary boxes with a scaling factor ratio:

(11) $$\begin{array}{c} w_{inner}=w\times ratio,\;\;h_{inner}=h\times ratio,\\ w_{inner}^{gt}=w^{gt}\times ratio,\;\;h_{inner}^{gt}=h^{gt}\times ratio, \end{array}$$

**Figure 8:**
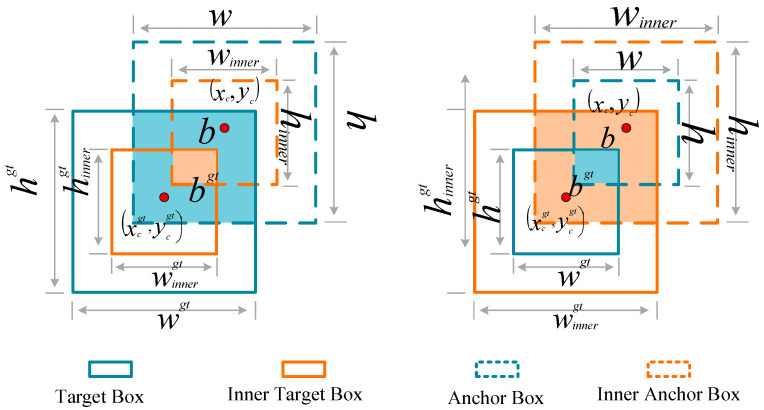
Description diagram of Inner_IoU.

In addition, by employing parallel computation of multi-scale auxiliary boxes, the proposed method not only maintains detection accuracy but also greatly improves model convergence speed, providing an effective solution for real-time object detection in complex scenarios. An illustration of the Inner-IoU is shown in Figure 8.

The Inner-IoU loss introduces adjustable auxiliary boxes, controlled by a scaling factor ratio, to optimize bounding box regression. When ratio < 1, the auxiliary box is shrunk, which increases the gradient magnitude and accelerates convergence for samples with high IoU. When *ratio* > 1, the auxiliary box is enlarged, enhancing regression performance for low-IoU samples.

(12) $$b_{i}^{gt}=x_{c}^{gt}-\frac{w^{gt}\times ratio}{2},\;b_{r}^{gt}=x_{c}^{gt}+\frac{w^{gt}\times ratio}{2},$$

(13) $$b_{t}^{gt}=y_{c}^{gt}-\frac{h^{gt}\times ratio}{2},\;b_{b}^{gt}=y_{c}^{gt}+\frac{h^{gt}\times ratio}{2},$$

(14) $$b_{1}=x_{c}-\frac{w\times ratio}{2},\;b_{r}=x_{c}+\frac{w\times ratio}{2},$$

(15) $$b_{t}=y_{c}-\frac{h\times ratio}{2},\;b_{b}=y_{c}+\frac{h\times ratio}{2},$$

The coordinates of the Inner Target Box and Inner Anchor Box are precisely calculated using Equations (12)–(15), and the final Inner-IoU loss is defined in Equation (18).

(16) $$inter=\left(\min\left(b_{r}^{gt},b_{r}\right)-\max\left(b_{1}^{gt},b_{1}\right)\right)*\left(\min\left(b_{b}^{gt},b_{b}\right)-\max\left(b_{t}^{gt},b_{t}\right)\right),$$

(17) $$union=\left(w^{gt}\times h^{gt}\right)*{\left(ratio\right)}^{2}+\left(w\times h\right)\times {\left(ratio\right)}^{2}-inter,$$

(18) $${IoU}^{inner}=\frac{inter}{union},$$

The gradient of Inner-IoU loss with respect to the original box parameters flows through the auxiliary boxes. Taking the derivative with respect to width *w*

(19) $$\frac{\partial L_{inner-IoU}}{\partial w}=\frac{{\partial L}_{inner-IoU}}{\partial w_{inner}}\times \frac{\partial w_{inner}}{\partial w}=\frac{\partial L_{inner-IoU}}{\partial w_{inner}}\times ratio,$$

The intersection area gradient is

(20) $$\frac{\partial_{inter}}{\partial w_{inner}}=\frac{\partial }{\partial w_{inner}}\left[\min\left(b_{r}^{gt},b_{r}\right)-\max\left(b_{l}^{gt},b_{l}\right)\right]\times H_{inter},$$

where $H_{inter}$ is the intersection height. The union area gradient is

(21) $$\frac{\partial_{union}}{\partial w_{inner}}=2\times ratio\times h_{inner}-\frac{\partial_{inter}}{\partial w_{inner}},$$

Applying the quotient rule for *IoU*:

(22) $$\frac{{\partial IoU}^{inner}}{\partial w_{inner}}=\frac{1}{{union}^{2}}\left(union\times \frac{\partial_{inter}}{\partial w_{inner}}-inter\times \frac{\partial_{union}}{\partial w_{inner}}\right),$$

When ratio<1, the auxiliary boxes are smaller, resulting in

(23) $$\begin{array}{c} {union}_{inner}=\left(w^{gt}\times h^{gt}\right)\times {ratio}^{2}+\left(w\times h\right)\times {ratio}^{2}-{inter}_{inner}\\ {union}_{inner}<\&{union}_{original}, \end{array}$$

This smaller union leads to larger IoU values for the same overlap, and consequently amplified gradients:

(24) $$\left|\frac{\partial L_{inner-IoU}}{\partial w}\right|\approx \frac{1}{{ratio}^{2}}\times \left|\frac{\partial L_{IoU}}{\partial w}\right|,$$

For ratio=0.75, the gradient amplification factor is $1/0.75^{2}\approx 1.78$, accelerating convergence for high-IoU samples without causing gradient explosion.

This formulation not only models the positional relationships of target boxes more accurately, thereby improving detection precision and localization performance, but also allows seamless integration with existing IoU-based methods such as GIoU and DIoU, enabling balanced optimization across different IoU scenarios.

## 4. Experimental Results and Analysis

### 4.1. Experimental Setup and Parameter Configuration

All experiments were conducted on a 64-bit Windows 11 operating system with an NVIDIA GeForce RTX 4080 GPU. The programming environment was Python 3.9, using the PyTorch 2.4.1 deep learning framework and CUDA version 11.6. The training parameters are shown in the Table 2.

### 4.2. Dataset Processing

**Figure 9:**
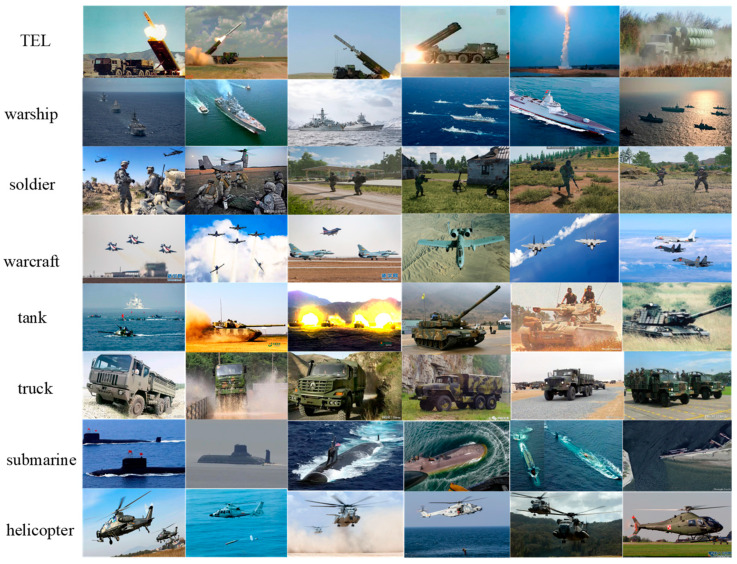
Sample Subset of the Dataset.

Due to the particularity of the application environment, datasets available for object detection are extremely scarce. Therefore, this paper constructs a dataset based on typical target units encountered in actual scenarios, with the scenes categorized into sea, land, and air. The basic target units can be divided into ships, submarines, tanks, infantry, fighter jets, helicopters, and so on. The dataset contains a total of 4197 images, with some sample images shown in Figure 9.

The construction of the dataset mainly involves data collection, data preprocessing, and data augmentation. Firstly, the dataset is collected based on commonly encountered target units in practical application scenarios, which are categorized into sea, land, and air. Basic target units include ships, submarines, tanks, infantry, fighter jets, helicopters, and so on. The study acquires raw data through various methods, such as searching and downloading from the internet, extracting key frames from exercise videos, and using web crawlers to collect relevant imagery data, in order to ensure the diversity of the data and the coverage of detection tasks.

Next, the collected data undergoes screening and annotation. Data of poor quality, excessive noise, or irrelevance is removed, and manual annotation is performed to ensure clear target categories and accurate annotation boundaries. After annotation, strict quality checks are conducted, correcting any erroneous annotations and ensuring consistency between image names and annotation names, thereby guaranteeing the accuracy and reliability of the data.

During the data augmentation phase, the study applies both failure-oriented image enhancement methods and environment-oriented style transfer techniques to expand the dataset. Failure-oriented image enhancement first employs conventional data augmentation methods, such as rotation, scaling, flipping, color adjustment, and contrast enhancement. Additionally, taking into account the characteristics of complex environments, composite image enhancement and multiple failure-mode image enhancement are applied to further expand the dataset. Environment-oriented style transfer techniques focus on transformations such as sunny → snowy, sunny → cloudy, sunny → foggy, and sunny → thunderstorm conditions.

Finally, all data undergo a second round of screening and quality review, removing data that may have annotation errors or suboptimal augmentation effects, to ensure that the resulting dataset is of high quality and consistency. At the same time, data formats are standardized, storage paths are normalized, and metadata management is improved to facilitate subsequent training and model optimization.

### 4.3. Evaluation Metrics

Evaluating the similarity between generated and real images is crucial in generative model assessment. In this study, FID, LPIPS, and SSIM were adopted to assess the quality of generated images:

FID (Fréchet Inception Distance) measures the statistical distance between real and generated image features extracted using the Inception v3 network. A lower FID indicates higher similarity between generated and real images.

LPIPS (Learned Perceptual Image Patch Similarity) quantifies perceptual similarity based on high-level semantic features learned by deep networks. A lower LPIPS value implies better perceptual similarity.

SSIM (Structural Similarity Index) evaluates image similarity from three aspects: luminance, contrast, and structure. A higher SSIM suggests greater structural consistency.

For object detection performance, mean Average Precision (mAP) and computational complexity (GFLOPs) were used. mAP reflects the model’s accuracy across different IoU thresholds—the higher the value, the better the detection performance. GFLOPs measures the number of floating-point operations required to process a single image—a lower value indicates better computational efficiency. Together, mAP and GFLOPs offer a comprehensive evaluation of model accuracy, efficiency, and deployment feasibility.

### 4.4. Comparative Experiments

#### 4.4.1. Improved CycleGAN Comparison

To validate the effectiveness of the improved model for style transfer, experiments were conducted on the summer-to-winter translation dataset provided by the official CycleGAN repository. The dataset includes 1231 training and 309 testing images for the summer domain, and 962 training and 238 testing images for the winter domain. The proposed method was compared with standard CycleGAN and DenseNet CycleGAN.

As shown in Table 3 (↓ indicates lower is better, ↑ indicates higher is better), our method consistently outperforms the baselines in the image style transfer task. The results are summarized as follows:

(1) FID: Our method achieves scores of 50.533 (summer → winter) and 60.351 (winter → summer), which are 26.163 and 13.670 lower than CycleGAN, and 27.846 and 16.282 lower than DenseNet CycleGAN, respectively.

(2) LPIPS: Our scores of 0.135 (summer → winter) and 0.138 (winter → summer) represent reductions of 0.044 and 0.013 over CycleGAN, and 0.046 and 0.038 over DenseNet CycleGAN.

(3) SSIM: Our method achieves 0.884 (summer → winter) and 0.886 (winter → summer). Although slightly lower than CycleGAN, these values are 0.011 and 0.025 higher than those of DenseNet CycleGAN.

The marginal decrease in SSIM is primarily due to structural changes resulting from brightness and detail adjustments during style transfer. However, the significant improvements in FID and LPIPS demonstrate substantial gains in perceptual quality.

Overall, both objective and subjective evaluations confirm that the proposed method preserves essential content while significantly improving visual naturalness and realism, yielding results that better match the target style and align more closely with human perceptual expectations. These enhancements demonstrate the method’s effectiveness for both style transfer and data augmentation applications.

#### 4.4.2. Ablation Experiment of the Improved CycleGAN

To verify the effectiveness of the proposed approach for style transfer, an ablation study was conducted based on the CycleGAN framework. We compared three configurations: (1) CycleGAN with dual self-attention, (2) CycleGAN with feature consistency loss (FC_LOSS), and (3) the proposed method, which integrates both modules. All models were trained under the same configuration, dataset, and evaluation metrics. Objective results for both summer-to-winter and winter-to-summer style transfer tasks are presented in Table 4.

The results (↓ denotes lower is better; ↑ denotes higher is better) demonstrate the following:

After adding dual self-attention to the transition task from summer to winter, the model’s performance on FID improved by 24.968, LPIPS improved by 0.036, and SSIM was 0.879, proving that dual self-attention significantly optimized perception metrics. The addition of FC_LOSS improved the performance of the model on FID by 9.870, LPIPS by 0.01, and SSIM by 0.022, demonstrating that FC_LOSS effectively enhances the fidelity of the structure. The method proposed in this article combines the excellent performance of self-attention on FID and LPIPS, and the excellent performance of FC_LOSS on SSIM, achieving optimal values of 50.533 and 0.135 on FID and LPIPS, respectively. Compared with simply adding a self-attention mechanism module, SSIM has also been improved.

In the transition task from winter to summer, adding Dual self-Attention improved the model’s performance on FID by 8.963, LPIPS by 0.009, and SSIM by slightly decreasing. After adding FC_LOSS, the model’s performance on FID improved by 3.581, LPIPS improved by 0.003, and SSIM improved by 0.011. The method proposed in this article achieved optimal values of 60.351 and 0.138 on FID and LPIPS, respectively, while reducing the fluctuation of SSIM.

These findings confirm that our approach consistently outperforms baseline CycleGAN and individual enhancements in both transfer directions. Particularly, significant gains in FID and LPIPS metrics, along with stable SSIM, highlight the method’s ability to jointly optimize perceptual quality and structural fidelity, satisfying practical application requirements.

**Figure 10:**
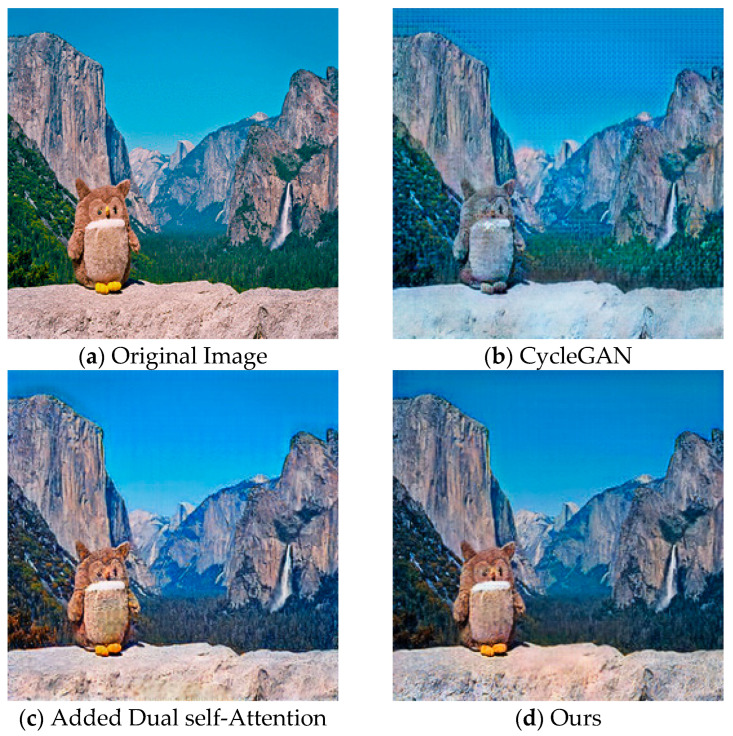
Generated Results Comparison Diagram. Figure (**a**) shows the original image, (**b**) shows the image generated by CycleGAN, (**c**) shows the image generated by CycleGAN with self-attention added, and (**d**) shows the image generated by our model.

As shown in Figure 10, the visual comparison for the summer-to-winter task reveals that, compared to CycleGAN (which produces noticeable artifacts and structural distortion), our method improves global context modeling and effectively suppresses artifacts through dual self-attention. The addition of FC_LOSS further enhances atmospheric consistency, generating a natural snowy haze that conforms to depth-based attenuation, while preserving foreground details. The combination of dual self-attention and FC_LOSS significantly improves texture clarity, foreground-background illumination balance, and overall scene realism, validating the proposed model’s effectiveness in style transfer tasks.

#### 4.4.3. Simulation Results Comparison

**Figure 11:**
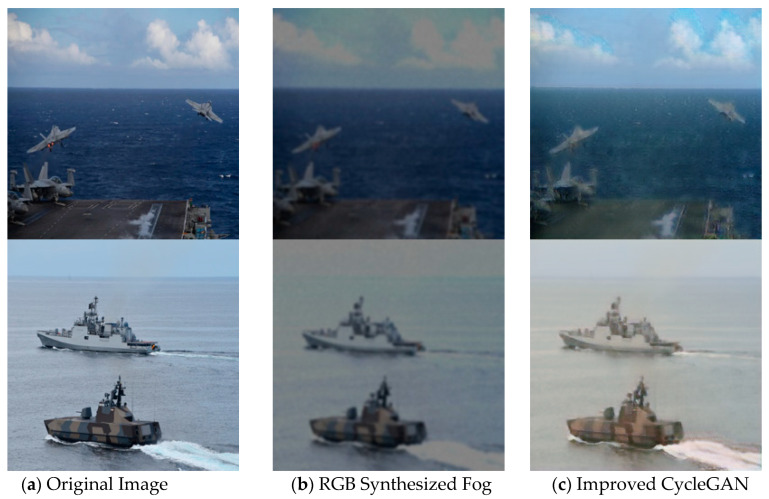
Effect comparison in maritime fog scenarios. (**a**) is the original image, (**b**) is the RGB synthesized fog image, and (**c**) is the improved CycleGAN synthesized fog image.

(1) Subjective Evaluation:

Figure 11 compares the generation effect of improved CycleGAN and traditional RGB synthesized fog. Figure 11a is the original image, Figure 11b is the RGB synthesized fog image, and Figure 11c is the improved CycleGAN synthesized fog image. The RGB-based method applies a uniform fog filter across the image without accounting for scene structure, resulting in visually flat fog effects that lack realism. The haze tends to obscure details and diminish visual depth. In contrast, the improved CycleGAN learns regional structure and texture differences dynamically, enabling realistic fog concentration gradients.

In maritime fog scenarios in particular, the foreground retains moderate haze that allows object contours to remain visible, while the background exhibits distance-based visibility decay. Furthermore, based on atmospheric scattering principles, the model adjusts global tones to cooler gray hues and suppresses excessive brightness contrast—better aligning with optical properties of real foggy scenes.

(2) Objective Evaluation:

Due to the fact that the comparison image samples are collected from the original dataset proposed in the article, the quality of the images themselves may differ compared to the data samples provided by CycleGAN. Therefore, various indicators of the images may be slightly affected, but it does not affect the comparison experiment.

The objective experimental results in Table 5 indicate that the improved CycleGAN has an FID value of 110.425, LPIPS value of 0.288, and SSIM value of 0.795 in fog image synthesis tasks. Compared to traditional RGB synthesis fog method, FID decreased by 8.135, LPIPS decreased by 0.096, and SSIM slightly decreased. The differences in sample sources may introduce slight fluctuations in indicators, but the improvement in perceived quality (FID/LPIPS optimization) validates the authenticity advantage of the model generation effect. The limited loss of SSIM is due to the emphasis on texture richness and physical authenticity, and overall, it still significantly improves the consistency of image perception.

#### 4.4.4. Comparison Experiment of the Improved YOLO

(1) Comparison experiment of feature fusion methods

To verify the effectiveness of the proposed AFPN (Asymptotic Feature Pyramid Network) within the YOLOv8n architecture, we conducted comparative experiments against mainstream feature fusion approaches. The results are presented in Table 6. The experimental findings indicate: FPN lacks a bottom-up fusion path, resulting in suboptimal multi-scale feature integration and limited detection accuracy; BiFPN, while enhancing performance through a weighted feature balancing mechanism, introduces significant computational overhead due to its complex structure; In contrast, AFPN achieves the best performance across key metrics, including mAP@0.5, mAP@0.95, and computational cost (FLOPs).

Although AFPN introduces slightly more parameters than other fusion modules, it incorporates feature dimension reduction techniques to achieve the lowest FLOPs among the compared methods. By maintaining the computational efficiency of PANet and adopting a more effective fusion strategy, AFPN simultaneously improves detection accuracy and efficiency, validating its effectiveness as a replacement for PANet in the YOLOv8n backbone.

(2) Comparison Experiments with Mainstream Detection Algorithms

To comprehensively evaluate the performance of the proposed AS-YOLO method in complex environments, the article compares it with multiple versions of the YOLO series and the Transformer-based RT-DETR algorithm. The comparison results are shown in Table 7. The main findings include: AS-YOLO achieves an mAP@0.5 of 83.0%, which is 1.8%, 2.8%, 2.3%, 1.5%, 2.1%, 3.8%, and 1.8% higher than YOLOv5n, YOLOv6n, YOLOv7n, YOLOv8n, YOLOv11n, YOLOv12n, and RT-DETR18, respectively; for mAP@0.95, AS-YOLO reaches 57.4%, which is 2.7%, 1.1%, 0.8%, 0.6%, 1.5%, and 3.7% higher than the corresponding YOLO series comparison models, respectively; although it is 0.3% lower than RT-DETR in the mAP@0.95 metric, RT-DETR has a computational cost of 58.3GFLOPS, which is 7.6 times that of AS-YOLO. These results confirm that the AS-YOLO model proposed in this article significantly improves detection accuracy while reducing computational complexity. Its outstanding performance and balanced efficiency in complex environments make it promising for practical applications.

#### 4.4.5. Ablation Experiment of YOLO

To systematically evaluate the performance improvement brought by each proposed module, we conducted ablation experiments based on the YOLOv8n baseline model by progressively integrating the c2f_CBAM module, AFPN feature pyramid, and Inner-IoU loss function.

As shown in Table 8, the experimental results demonstrate the following: directly applying CBAM to the backbone increases computational cost without noticeable improvement in detection performance; however, incorporating CBAM into the deep c2f modules improves mAP@0.5 and mAP@0.95 by 0.4% and 0.3%, respectively. This indicates that the c2f_CBAM module significantly enhances the model’s attention to small objects and their Introducing the AFPN module into the neck improves mAP@0.5 and mAP@0.95 by 0.6% and 0.9%, while reducing computational overhead through feature dimension reduction, achieving the lowest FLOPs among all configurations; Finally, integrating the Inner-IoU loss function further boosts mAP@0.5 and mAP@0.95 by 1.5% and 0.6%, respectively, compared to the baseline. These results validate the effectiveness of the synergistic optimization of all three modules, particularly in challenging small object detection scenarios.

To validate generalization across different object categories, we provide detailed per-class AP comparison between baseline YOLOv8n and AS-YOLO. Table 9 presents the results for all nine target categories.

(1) Consistent Overall Improvement: AS-YOLO improves overall mAP@0.5 from 81.5% to 83.0%, demonstrating robust generalization across diverse target categories.

(2) Largest Gains for Challenging Classes: The most significant improvements are observed for warcraft (+4.5%), submarine (+3.9%), and warship (+2.3%). These categories typically involve challenges such as partial occlusion (submarine below water surface), complex aerial backgrounds (warcraft), and scale variations (warship at different distances). The improvements validate that AFPN’s multi-scale fusion and c2f_CBAM’s attention mechanism effectively address these challenges.

(3) Stable Performance for Well-Performing Classes: Categories with already high baseline performance (tank: 93.6%, helicopter: 91.1%, truck: 90.2%) maintain or slightly improve their AP, indicating no negative transfer effects.

(4) Trade-off Analysis: The slight decrease in TEL (-1.6%) suggests a minor trade-off in feature allocation when optimizing for overall performance. This class-specific variation indicates potential for future work on adaptive, class-aware feature weighting.

**Figure 12:**
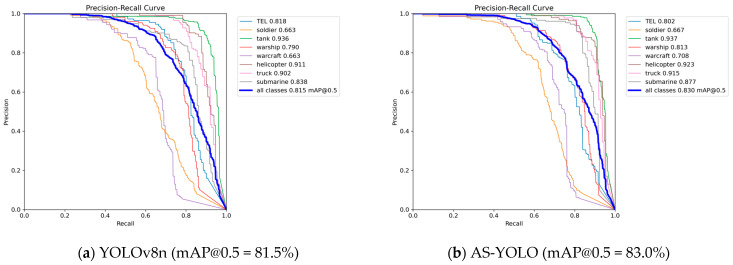
Precision-Recall Curve Comparison. (**a**) shows the P-R curve of YOLOv8n, (**b**) shows the P-R curve of AS-YOLO.

Figure 12 presents the precision-recall curves for both models.

The P-R curves demonstrate that AS-YOLO maintains higher precision at equivalent recall levels for most categories, particularly in the high-recall region, indicating improved handling of difficult samples and better confidence calibration.

**Figure 13:**
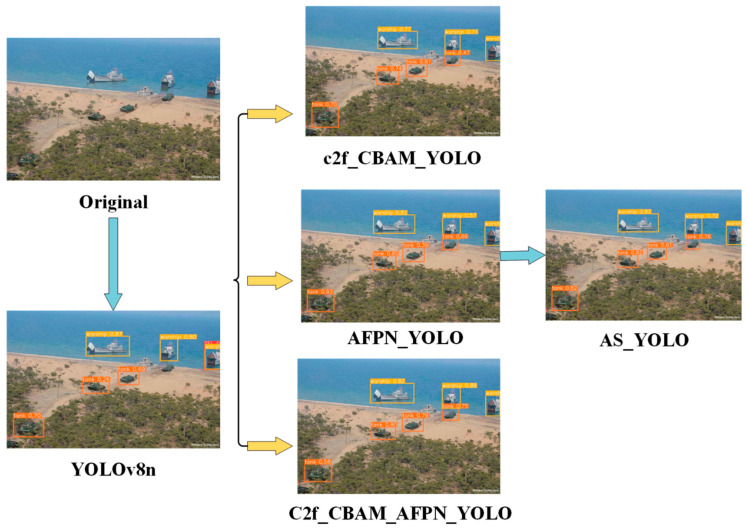
Diagram of the test results of each module.

Figure 13 provides a visual comparison of detection results. Unlike the baseline YOLOv8n model—which suffers from false positives and missed small targets—the proposed model leverages spatial-channel attention to eliminate false detections. The AFPN module enhances feature representation under occlusion, resulting in notable improvements in detecting occluded targets. When all three modules are combined, the model exhibits superior performance in cluttered backgrounds and small-object scenarios, proving the practical applicability and robustness of the proposed AS-YOLO architecture.

Overall, the AS-YOLO model achieves a 1.5% improvement in total mAP compared to the baseline YOLOv8n, mainly attributed to structural enhancements that strengthen feature extraction and enable finer-grained target discrimination. These improvements validate the effectiveness and generalizability of the proposed method in real-world applications.

#### 4.4.6. Visualization Analysis

**Figure 14:**
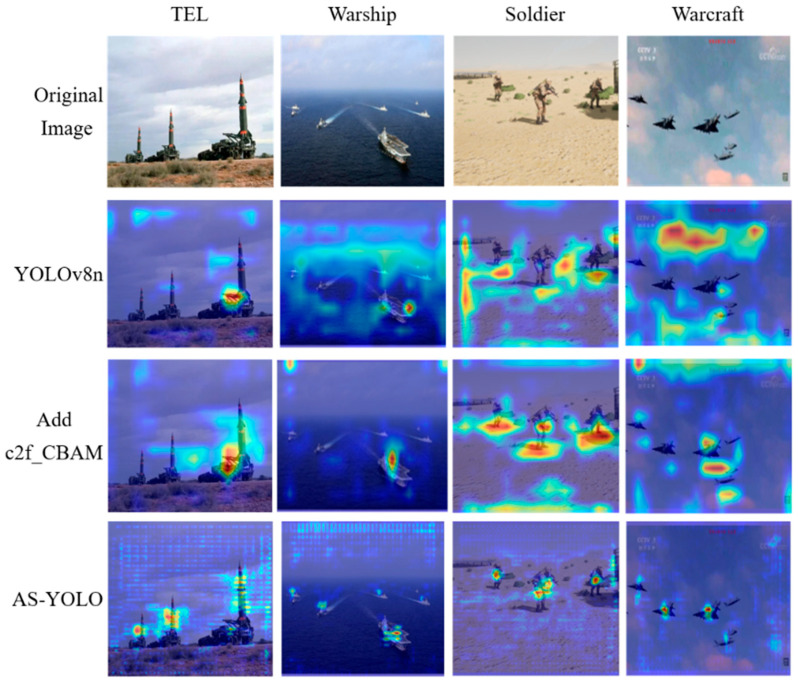
Visualization heat maps of different modules.

To further interpret the model’s behavior, Grad-CAM [35] was employed for visual analysis. As illustrated in Figure 14, the AS-YOLO model with the c2f_CBAM module exhibits more accurate and concentrated activation regions compared to YOLOv8n. The attention mechanism allows the network to focus more precisely on the actual locations of target objects, thereby significantly increasing detection accuracy. Moreover, the model can adaptively assign importance weights to different target regions, focusing more on essential features of the object rather than background noise.

This improvement is especially pronounced in small object detection tasks, where the refined focus of activation regions leads to substantial gains in accuracy, demonstrating the effectiveness of the c2f_CBAM module in enhancing target feature representation.

#### 4.4.7. Comparison Experiment of Data Augmentation

To improve dataset diversity under complex environmental conditions, weather style transfer was performed using the proposed enhanced CycleGAN. Considering that foggy conditions dominate real-world maritime and adverse environments, we assigned the following augmentation ratios for synthetic weather styles: snow: fog: thunderstorm: cloudy = 1:3:1:1. The expanded dataset contains 12,591 image samples from complex weather environments. The training, validation, and testing sets of the data samples were divided in a ratio of 7:2:1. Table 10 shows the specific number of style transfer enhanced data.

This study systematically verifies the effectiveness of the proposed style transfer-based image augmentation strategy in improving object detection performance under complex environments. The experimental setup includes two stages: (1) training both the baseline YOLOv8n and the proposed AS-YOLO models on the original dataset, and (2) training the same models on the style-transferred augmented dataset, followed by evaluation on a complex environment test set.

As shown in Table 11, the results indicate that the style transfer augmentation strategy significantly improves detection performance for both models. For YOLOv8n, mAP@0.5, mAP@0.75, and mAP@0.95 increased by 17.2%, 14.3%, and 18.0%, respectively. For the improved AS-YOLO, these metrics improved by 14.6%, 14.5%, and 17.8%, respectively, achieving final scores of 96.2%, 82.8%, and 79.4%.

Compared to the baseline, the improved model consistently outperformed on both the original and augmented datasets, with particularly notable gains under higher IoU thresholds. This validates that the proposed method not only enhances the model’s adaptability to varying lighting and weather conditions but also significantly boosts its robustness in high-precision detection tasks, demonstrating the practical value and application potential of style-transfer-based data augmentation in real-world complex scenarios.

**Figure 15:**
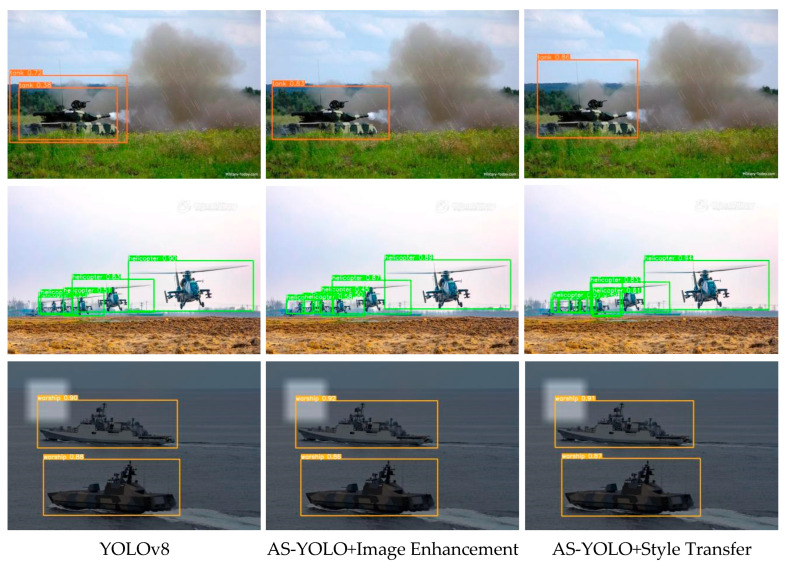
Visualization results in different complex environments.

To more intuitively demonstrate the effectiveness of the proposed image enhancement for object detection, Figure 15 presents the visualization comparison results of images under different complex environments. The first column shows the detection results of YOLOv8, the second column shows the detection results of the AS-YOLO model with failure-oriented image enhancement added, and the third column shows the detection results of the AS-YOLO model with style transfer image enhancement added. As shown in Scene 1, due to smoke interference and target occlusion in the picture, the base model made a misjudgment, detecting the distant mountain as a tank with a similar color. In Scene 2, the mutual occlusion among multiple targets led to missed detections by the base model. The detection accuracy of the AS-YOLO model trained with data augmentation was improved. Further, the AS-YOLO model after style transfer failure enhancement achieved even better detection results. Similarly, in Scene 3, under the influence of weather interference, the enhanced AS-YOLO models performed better than the base model in terms of detection performance.

#### 4.4.8. Generalization Experiment

To verify the generalization and robustness of the proposed method in this paper, the WSODD dataset was selected for generalization experiments to demonstrate the application potential of the AS-YOLO framework in other complex environments. The WSODD dataset consists of 7467 water surface images captured by Hikvision, with a resolution of 1920 × 1080 for each image. It includes various environments such as oceans, lakes, and rivers, as well as different lighting conditions like day, dusk, and night, and different weather conditions like sunny, cloudy, and foggy. The dataset has 14 categories (bridge, ship, boat, ball, rubbish, rock, buoy, platform, harbor, mast, tree, animal, grass, person), with a total of 21,911 target instances. In this paper, the image samples were divided into training, validation, and test sets in a 7:2:1 ratio.

The results of the generalization experiments are shown in Table 12. Compared with the detection results of the base model YOLOv8n, AS-YOLO improved the mAP0.5 and mAP0.95 metrics by 2.1% and 1.3%, respectively. After combining the style transfer image enhancement method proposed in this paper, AS-YOLO achieved mAP0.5 and mAP0.95 of 83.7% and 50.2%, respectively, with improvements of 7.8% and 7.4%. This fully validates that the image enhancement method proposed in this paper can enhance the robustness and accuracy of the model in complex sea surface scenarios, and also proves the excellent generalization performance of the proposed method.

## 5. Conclusions

This paper proposes an innovative framework for joint optimization of image augmentation and object detection, aimed at improving detection performance in complex environments through a dual-path strategy. On the image augmentation side, an adaptive style transfer algorithm based on an improved CycleGAN architecture is developed. It achieves high-fidelity image enhancement tailored to diverse weather and lighting conditions, significantly expanding the coverage of the training dataset. On the detection model side, an enhanced AS-YOLO detector is built upon the YOLOv8 backbone. By integrating the CBAM attention mechanism, the AFPN, and the Inner-IoU loss function, a more accurate feature extraction–fusion–localization pipeline is constructed.

Extensive experiments confirm the effectiveness and adaptability of the proposed augmentation technique and model design under complex backgrounds. Despite the promising results in improving detection accuracy, some limitations remain. First, the physical realism and stylistic diversity of the generated images could be further improved. Second, detailed quantitative analysis of failure cases—including false positive categorization, size-stratified miss rates, and occlusion sensitivity—was not conducted in this study, which would provide deeper insights into the model’s robustness boundaries under extreme conditions. Future work will focus on the following four directions: (1) Developing physically based generative models to improve image realism; (2) Designing a continuous, controllable style transfer framework guided by environmental parameters; (3) Exploring light-weight end-to-end architectures that unify detection and augmentation, to facilitate deployment in real-world scenarios; and (4) Conducting comprehensive failure mode analysis to systematically evaluate robustness under varying object scales, occlusion levels, and adverse weather conditions.

## Figures and Tables

**Table 1:** Feature Extraction Layer Specifications.

Layer Index	VGG-19 Layer	Feature Level	Channels	Spatial Size	Captured Information
1	relu1_2	Shallow	64	H × W	Edges, textures
2	relu3_4	Intermediate	256	H/4 × W/4	Patterns, local structures
3	relu5_4	Deep	512	H/16 × W/16	Semantic content, objects

**Table 2:** Training Parameters.

Parameter	Value
ImageSize	640
epochs	200
Batch Size	16
warmup	8
Learning Rate	0.01
Optimizer	SGD
Momentum	0.937

**Table 3:** Comparison Experiment.

	Method	FID↓	LPIPS↓	SSIM↑
	CycleGAN	76.696	0.179	0.890
Summer → Winter	DenseNet CycleGAN	78.379	0.181	0.873
	Ours	50.533	0.135	0.884
	CycleGAN	74.021	0.151	0.912
Winter → Summer	DenseNet CycleGAN	76.633	0.176	0.861
	Ours	60.351	0.138	0.886

**Table 4:** Ablation Experiment.

	Method	FID ↓	LPIPS ↓	SSIM ↑
Summer → Winter	CycleGAN	76.696	0.179	0.890
	Dual self-attention	51.728	0.143	0.879
	FC_LOSS	66.826	0.169	0.912
	Ours	50.533	0.135	0.884
Winter → Summer	CycleGAN	74.021	0.151	0.892
	Dual self-attention	65.058	0.142	0.875
	FC_LOSS	70.44	0.148	0.903
	Ours	60.351	0.138	0.886

**Table 5:** Objective Evaluation. ↓ denotes lower is better; ↑ denotes higher is better.

Method	FID ↓	LPIPS ↓	SSIM ↑
RGB synthesis fog method	118.56	0.384	0.814
Improved CycleGAN	110.425	0.288	0.795

**Table 6:** Comparison experiment of feature fusion methods.

Method	mAP_0.5_/%	mAP_0.95_/%	FLOPs/G
FPN	81.0	56.3	8.0
PAFPN	81.5	56.8	8.1
BiFPN	81.8	56.2	8.4
AFPN	82.1	56.9	7.2

**Table 7:** Comparative experiments with mainstream detection algorithms.

Method	mAP_0.5_/%	mAP_0.95_/%	FLOPs/G
YOLOv5	81.2	54.7	7.1
YOLOv6	80.2	56.3	11.8
YOLOv7	80.7	56.6	20.3
YOLOv8	81.5	56.8	8.1
YOLOv11	80.9	55.9	6.6
YOLOv12	79.2	53.7	6.7
RT-DETR R18	81.2	57.7	58.3
AS-YOLO	83.0	57.4	7.7

**Table 8:** Results of ablation experiments.

Method	mAP_0.5_/%	mAP_0.95_/%	FLOPs/G
YOLOv8n	81.5	56.8	8.1
+ CBAM	81.0	56.6	8.2
+ c2f_CBAM	81.9	57.1	8.1
+ AFPN	82.1	57.7	7.2
+ c2f_CBAM AFPN	82.4	56.9	7.7
AS-YOLO	83.0	57.4	7.7

**Table 9:** Per-Class Detection Performance Comparison.

Class	YOLOv8n/%	AS-YOLO/%	ΔAP/%
TEL	81.8	80.2	−1.6
soldier	66.3	66.7	+0.4
tank	93.6	93.7	+0.1
warship	79.0	81.3	+2.3
warcraft	66.3	70.8	+4.5
helicopter	91.1	92.3	+1.2
truck	90.2	91.5	+1.3
submarine	83.8	87.7	+3.9
Mean	81.5	83.0	+1.5

**Table 10:** Style transfer enhanced data distribution.

	Snow	Fog	Cloudy	Thunderstorm
Number of images	1399	4197	1399	1399

**Table 11:** Data Augmentation Comparison Experiment.

Method	mAP_0.5_/%	mAP_0.75_/%	mAP_0.95_/%
YOLOv8n	77.6	66.0	58.9
AS_YOLO	81.6	68.3	61.6
YOLOv8n + Style Transfer	94.8	80.3	76.9
AS-YOLO + Style Transfer	96.2	82.8	79.4

**Table 12:** Comparative experiment of generalization.

Method	mAP_0.5_/%	mAP_0.95_/%	FLOPs/G
YOLOv8n	73.8	41.5	8.5
AS-YOLO	75.9	42.8	8.1
AS-YOLO + Style Transfer	83.7	50.2	8.1

## Data Availability

The data presented in this study are openly available in GitHub at https://github.com/paofan666/Improved-CycleGAN (accessed on 1 November 2025).
